# A Case Report of Nephrotic Syndrome While Undergoing Quinine Therapy

**DOI:** 10.7759/cureus.2283

**Published:** 2018-03-07

**Authors:** Brittany Albrecht, Shelley Giebel, Michelle McCarron, Bhanu Prasad

**Affiliations:** 1 College of Medicine, University of Saskatchewan; 2 Department of Nephrology, Regina General Hospital; 3 Research and Performance Support, Saskatchewan Health Authority

**Keywords:** minimal change disese, nephrotic syndrome, adverse effects, quinine, adverse reaction

## Abstract

We summarize the case of an 81-year-old Caucasian female who presented to her family physician with signs and symptoms of nephrotic syndrome following a brief exposure to quinine. Prior to that visit, she was clinically well with no chronic medical ailments and met with her family physician for annual physical assessments. She had taken 11 tablets of quinine for nocturnal leg cramps over the course of 28 days before starting to notice mild peripheral edema, which subsequently progressed, leading to a family physician review. Her initial serum albumin level was 12 g/L, and a 24-hour urine protein output was quantified at 8.14 g/day; she was diagnosed as having nephrotic syndrome. A kidney biopsy confirmed the diagnosis of minimal change disease (MCD). Quinine therapy was stopped, and she was initiated on a tapering regime of prednisone with concurrent cyclosporine therapy. Within a fortnight of starting therapy, she went into remission and her immunosuppressive medications were rapidly tapered and discontinued. This paper reports an association between the use of quinine and subsequent MCD. This case report proposes that the use of quinine has an association with, and may be causal for, the development of minimal change disease. As this is yet an unreported adverse effect, this paper seeks to increase the knowledge of the varied and numerous effects of quinine.

## Introduction

The pharmacotherapeutic use of quinine has been predominantly associated with thrombocytopenia and thrombotic microangiopathy [1]. Nephrotic syndrome as a consequence of minimal change disease (MCD) has thus far not been associated with quinine use. MCD has been associated with a number of medications, including non-steroidal anti-inflammatories (NSAIDs), cyclooxygenase (COX-2) inhibitors, ampicillin, lithium, pamidronate, and sulfasalazine [2]. In this case report, we report nephrotic syndrome, potentially secondary to quinine use, in an elderly woman who was in a premorbid state of excellent health.

## Case presentation

An 81-year-old retired nurse was prescribed quinine by her family physician for recent onset nocturnal leg cramps. She was otherwise well, did not take any prescription medications, and met her physician only for yearly review. She claimed to have taken 11 quinine tablets over a 28-day period when she noticed peripheral edema, which became worse over the ensuing 48 hours, leading to a visit with the family doctor. On initial clinical review, she denied any other prior history of diabetes, hepatitis B, C, or human immunodeficiency virus (HIV). She also denied any history of exposure to NSAIDs, history of allergies, weight loss, anorexia, or any noticeable lymphadenopathy. Physical examination findings were positive for significant peripheral edema and negative for generalized lymphadenopathy. The cardiovascular exam was unremarkable with a blood pressure of 138/72 mm Hg and normal heart sounds. The lungs were clear, and abdominal examination revealed no evidence of ascites or palpable organomegaly. She had evidence of peripheral edema.

Initial lab results revealed the presence of protein, but no blood on urine analysis. Her serum urea was 6.5 mmol/L, creatinine 65 umol/L, and total cholesterol was 11.20 mmol/L. Serum albumin was 12 g/L, spot urine albumin/creatinine (ACR) was 1,012.4 (mg/mmol), and on quantification, there was 8.14 g/day of protein in the urine. These results were consistent with a clinical diagnosis of nephrotic syndrome.

Other relevant investigations were as follows: antinuclear antibody (ANA) - negative, anti-myeloperoxidase and proteinase 3 (PR3) - negative, anti GBM - negative, C3 (g/L) - 1.83 (0.74 - 1.85), and C4 (g/L) - 0.35 (0.44 - 1.16). Serum immunoglobulin levels included IgA (g/L): 6.35 (0.87-) and IgG (g/L): 6.24 (5.5 - 17.4). Serum protein electrophoresis (SPEP) revealed a polyclonal rise in immunoglobulins, but there was no identifiable monoclonal protein on immunofixation. We failed to identify cryoglobulins after three days of refrigeration. She was negative for hepatitis B, C, and HIV.

Due to the rapid onset of nephrotic syndrome, she was sent for an urgent kidney biopsy. The kidney biopsy on light microscopy yielded three cores of 19 glomeruli, seven of which were globally sclerotic. There was a mild focal increase in the matrix without an increase in cellularity and no endothelial swelling. There were no deposits in the mesangial, subendothelial, or subepithelial space. On immunofluorescence, there were four glomeruli, which showed no evidence of staining with immunoglobulin or complement. On electron microscopy, there were no deposits. However, there was diffuse effacement of the foot processes; these changes were consistent with minimal change disease.

The patient was convinced that the quinine had led to her symptoms and discontinued the medication. She was initiated on furosemide at a dose of 120 mg/day, 1 mg/kg body weight of prednisone with a tapering regime over four months, atorvastatin, 40 mg a day, risedronate, 35 mg per week, aspirin pending a kidney biopsy, and fluid-restricted to 1.5 l/day; it was also suggested that she follow a salt-restricted diet. In addition, the patient was started on 1 mg/kg body weight cyclosporine twice a day as an adjunctive therapy to minimize the exposure of steroids. She responded immediately post-initiation of therapy, and the sequential lab values are documented in Table 1. Due to her excellent renal response, immunosuppression was rapidly tapered and stopped within four months; three years later, she continues to be in remission and leads an independent life.

**Table 1:** Treatment and Response Trending of the patient's quantitative response to treatment type and dosing shows sequential improvement in the patient’s renal status. bid: twice a day; D/C: discontinued

	Biopsy	+ 1 month	+2 months	+4 months	+5 months	+7 months	+12 months
U protein (g/24 hours)	8.14	0.21	0.03	0.15	0.19	0.04	0.12
Serum creatinine (mmol/L)	65	70	68	56	70	65	68
Prednisone dose in mg/day	70	30	20	5	D/C	D/C	D/C
Cyclosporine dose in mg (bid)	75 mg	50 mg	25 mg	25 mg	D/C	D/C	D/C

## Discussion

Primary MCD is usually a disease of children and young adults. In a retrospective series of 95 patients with MCD by Waldman et al., the mean age and standard deviation (SD) at onset in adults were 45 (1.6) years [3]. The mean and SD albumin were 22.1 (0.8), 42% had hypertension, and 18% had acute kidney injury at presentation. Seventy-three percent of the patients relapsed at 21.6 ± 9.6 weeks after discontinuation of therapy. Four patients progressed to end-stage renal disease (ESRD). Certainly, the outcomes in adults are different than in the pediatric group, where the relapse rate is lower. Similarly, a review by Tse et al. noted that older patients 72 ± 6.8 years had a higher prevalence of hypertension and lower creatinine clearance [4].

The pathogenesis of primary MCD is currently unclear. Primary MCD is associated with T-cell activation and the resultant cytokine or “permeability factor” mediated injury to the glomerular epithelial cells has been proposed as a major contributor to disease pathogenesis [5]. Secondary MCD occurs when the precipitant directly or indirectly results in the characteristic changes in permselectivity and morphology of the glomerular epithelial cells. Secondary MCD has been associated with many causes, including neoplasms (hematologic malignancies), infections (syphilis, tuberculosis, mycoplasma), medications (NSAID’s, penicillin, cephalosporin’s, pamidronate, sulfasalazine, and gamma interferon), allergy (house dust, medusa and bee stings, grass pollen), and occasionally associated with other systemic illness like systemic lupus erythematosus (SLE) and HIV [6].

Quinine is amongst the medications with the most adverse reactions. The organ system involvement includes hematologic (thrombocytopenia, neutropenia, thrombotic microangiopathy), dermatologic (eczema, dermatitis), systemic (fever, chills, hypotension), neurologic (stupor, obtunded), pulmonary (hypoxemia), endocrine (hypoglycemia), and musculoskeletal reactions (rhabdomyolysis). The kidneys are typically involved as a consequence of thrombotic microangiopathy (TMA) and occasionally interstitial nephritis [7]. For a medication to cause MCD, there needs to be a systemic release of permselectivity-promoting factors from activated inflammatory or immune cells or the breakdown products of the medication have a direct toxic effect on the integrity of the glomerular epithelial cells, leading to abnormal glomerular permeability [6].

Quinine has been commonly studied to understand the molecular mechanisms of drug-dependent antibody formation and is also a common cause of drug-induced disorders [8]. Quinine may have unique amphipathic properties that allow it to become integrated into complementarity determining regions of naturally occurring antibodies, creating a hybrid paratope that greatly increases binding affinity to cell surface antigens [9]. Quinine may also integrate into quasi-stable regions of cell surface integrins to alter the conformation of a target epitope, causing increased binding affinity of the naturally occurring antibodies [10].

While the above-mentioned mechanisms might trigger hematologic outcomes like thrombocytopenia, anemia, and neutropenia (due to drug-dependent antibodies attaching to platelets, red cells, and neutrophils), it does not explain the “permeability factor” mediated injury to the glomerular epithelial cells. However, our patient was exposed to quinine for a month, her symptoms resolved within 14 days of discontinuation, and the clinical remission happened far earlier than would be expected with primary MCD. Also, our patient was normotensive at presentation, there was no decline in the glomerular filtration rate (GFR) compared to baseline values, and there were no relapses four years after the initial presentation despite a relapse rate of 70% in adults [8]. Our patient’s presentation was in stark contrast to the reported presentation in the elderly. While re-exposure of the patient to the medication would have addressed the cause of the nephrotic syndrome, we did not explore the idea on ethical grounds.

## Conclusions

Our paper illustrates the correlation between quinine use and the development of minimal change disease. Though causation cannot be proven, this report seeks to increase the knowledge of the varied and numerous effects of quinine.
